# Traditional Chinese medicine prescriptions (XJZ, JSS) ameliorate spleen inflammatory response and antioxidant capacity by synergistically regulating NF-κB and Nrf2 signaling pathways in piglets

**DOI:** 10.3389/fvets.2022.993018

**Published:** 2022-09-16

**Authors:** Jian Chen, Nianqing Hu, Yaqing Mao, Aiming Hu, Wenjuan Jiang, Aimin Huang, Yun Wang, Puyan Meng, Mingwen Hu, Xiaobin Yang, Yuandong Cao, Fan Yang, Huabin Cao

**Affiliations:** ^1^Jiangxi Provincial Key Laboratory for Animal Health, Institute of Animal Population Health, College of Animal Science and Technology, Jiangxi Agricultural University, Nanchang, China; ^2^MOA Center for Veterinary Drug Evaluation, China Institute of Veterinary Drug Control, Beijing, China; ^3^Jian Animal Husbandry and Veterinary Bureau, Jian, China; ^4^Animal Husbandry and Aquatic Products Technology Application Extension Office, Jiangxi Agricultural Technology Extension Center, Nanchang, China; ^5^Jiangxi Vocational College of Technology, Nanchang, China; ^6^Jiangxi Academy of Forestry, Nanchang, China; ^7^Jiangxi Zhongchengren Pharmaceutical Co., Ltd., Nanchang, China; ^8^Jiangxi Jiabo Biological Engineering Co., Ltd., Jiujiang, China

**Keywords:** weaning stress, spleen function, traditional Chinese medicine, antioxidant capacity, anti-inflammatory

## Abstract

Weaning transition generally impairs the immune system, inducing immune disturbance, which may be associated with post-weaning diarrhea and high mortality in piglets. The spleen is a pivotal lymphatic organ that plays a key role in the establishment of the immune system. Traditional Chinese medicine (TCM) prescriptions, XiaoJianZhong (XJZ) and Jiansananli-sepsis (JSS), are widely used prescriptions for treating spleen damage and diarrhea. Here, we hypothesized that XJZ and JSS maintain the spleen physiological function by ameliorating antioxidant capacity and inflammatory response in weaned piglets. In this study, 18 weaned piglets were assigned to the Control, XJZ and JSS groups. By hematoxylin and eosin staining, hematological analysis, flow cytometric analysis, qRT-PCR and western blot, the effects of both TCM prescriptions on the spleen antioxidant defense system and inflammatory pathway were explored. Results showed that both TCM treatment significantly ameliorated the weaning-induced morphological damage in piglets, as evidenced by clearer and more perfect spleen histology, as well as higher relative area of white pulp. Meanwhile, both XJZ and JSS exerted better blood parameters, as supported by the changes of monocyte level and lymphocyte subpopulations CD4+/CD8+ ratio. Furthermore, the levels of inflammatory markers, IL1β, IL6, IL8, and TNF-α in the spleen were markedly decreased after supplemented with both TCM prescriptions. Importantly, the inhibition of nuclear factor-kappaB (NF-κB) and its downstream effector genes (IL6, IL8, and TNF-α) in both XJZ and JSS treatment groups further confirmed alleviation of inflammatory responses in the spleen. In addition, both XJZ and JSS enhanced the antioxidant capacity of the spleen by activating the nuclear factor erythroid 2-related factor 2 (Nrf2)-activated antioxidant defense system. Notably, the results of PCA and network correlation analysis indicated that XJZ and JSS treatment altered the expression profiles of inflammatory and antioxidant-related factors in the spleen of weaned-piglets, which may involve the synergy of NF-κB and Nrf2 signaling pathways. In summary, our study showed that TCM prescriptions, XJZ and JSS could ameliorate inflammatory response and antioxidant capacity in the spleen by synergistically regulating NF-κB and Nrf2 signaling pathways in piglets.

## Introduction

Weaning stress could impair immune system development and intestinal barrier function in piglets, resulting in growth inhibition, secondary infection, diarrhea and even death (1). Meanwhile, due to its weak immune and digestive system in weaned piglets, once the potential diseases are induced by stress, it will not only cause economic losses, but also damage animal welfare (2). Diarrhea and stress-induced secondary bacterial or viral infections are leading causes of mortality in weaned piglets. Hence, improving the immunity of piglets may be a feasible method to against weaning stress (3, 4). In the past decades, antibiotic, as a feed additive, has been employed in increasing daily gain and preventing disease of piglets or other economical animals (5, 6). Likewise, it also plays an important role in bacterial diarrhea control (6). However, the abuse of antibiotics has led to the multi-antibiotic and resistance antibiotic residue (7, 8). Many countries or regions, such as the United States, China, and European Union, have clearly stipulated that the use of antibiotics as feed additives is prohibited. Crucially, the antibiotics cannot fundamentally ameliorate the immune state of piglets, but affect the colonization and proliferation of normal or beneficial gut microflora (9). Therefore, it is imperative to find an alternative medicine with low toxicity, few side effects, and high efficiency.

Traditional Chinese medicine (TCM), derived from natural plants, has been shown to exert immune-potentiating efficacy (10). It has almost no side effects, which effectively solves the adverseness caused by antibiotics application. Importantly, multiple active compounds in TCM prescription could hit various targets and exert synergistic therapeutic efficacy (11). As classic TCM prescriptions, XiaoJianZhong (XJZ) and Jiansananli-sepsis (JSS) prescriptions have been used in China and Asian countries for hundreds of years, and they have been proven to boost immunity and treat diarrhea. However, the mechanism is still not fully understood. Previous study showed that both XJZ and JSS could increase the growth performance and control diarrhea induced by weaning in piglets (9, 12, 13). Here, we speculated that the alleviation of weaning-induced growth inhibition and diarrhea by XJZ and JSS is related to the improved spleen development in piglets. It is well-known that the spleen, as largest secondary immune organ, is responsible for initiating immune response to blood-borne antigens in the body, and filtering non-self-substances and damaged cells from the blood (14). Meanwhile, the spleen is a storage organ for T lymphocytes (Lym), which provides protection against pathogens and tissue damage (15). Maintenance of normal spleen function is extremely important for growth and development of young animal as it is involved in the maturation of digestive and immune processes (16). However, the spleen is sensitive to stress, inducing inflammatory responses and oxidative damage. It is well-known that nuclear factor-kappaB (NF-κB) and nuclear factor erythroid 2-related factor 2 (Nrf2) are transcription factors that regulate genes responsible for inflammatory and anti-oxidant response, respectively. NF-κB signaling and its subsequent pro-inflammatory cytokine expression, oxidative stress, apoptotic and anti-apoptotic genes play a critical role in stress-induced spleen injury. Recent studies have shown that weaning-induced inflammatory response and oxidative stress are the main causes of spleen damage in piglets (17, 18). Therefore, the main purpose of this study was to investigate the effects of TCM prescription, XJZ and JSS on spleen morphology, inflammatory response and antioxidant defense system in weaned piglets to test our above hypothesis.

Our results found that both XJZ and JSS could ameliorate blood parameters, relieve spleen morphological damage, elevate the peripheral blood Lym subpopulations CD4+/CD8+ ratio, alleviate inflammation and improve antioxidant capacity in the spleen of weaned-piglets by synergistically regulating NF-κB and Nrf2 signaling pathways.

## Materials and methods

### Animals, experimental design and rations

The experiment was performed at JiangXi Agricultural University's Researching and Teaching Base lasted 60 days. All experimental protocols were approved by the Committee for the Care and Use of Experimental Animals, Jiangxi Agricultural University, Jiangxi, China. Eighteen weaned-piglets (17.37 ± 1.32 kg, Large White × Landrace × Duroc) at 40 days of age were randomly divided into three groups (*n* = 6) according to weight and sex. The three experimental groups were fed with the same basic diet, and it was designed to meet the nutritional level of the piglets ((19), Supplementary Table 1). Experimental groups included: (I) Control group (basal diet), (II) XJZ group (basal diet plus 10 g/kg XJZ prescription), or (III) JSS group (basal diet plus 3 g/kg JSS prescription). The doses for TCM groups (XJZ and JSS) were determined based on our preliminary tests and previous study (9). All raw materials for XJZ and JSS prescriptions were provided by The Spirit Jinyu Biological Pharmaceutical Co., Ltd (Huhhot, Inner Mongolia, China). All dried Chinese herbs were smashed through a 2.5 mm screen sieve. Composition and main active constituents of XJZ and JSS are presented in Table 1.

**Table 1:** Composition and main active constituents of XJZ and JSS (air-dried basis)^a^.

**Scientific name**	**Main active ingredients**	**Proportion (%)**
**XJZ prescription**
*Cassia twig*	Cinnamaldehyde	13
*Glycyrrhiza uralensis*	Glycyrrhizin	4
*Ziziphus zizyphus*	*Jujuba* polysaccharide	4
*Cynanchum otophyllum*	Paeoniflorin	13
*Zingiber officinale Roscoe*	Ginger oleoresin	6
*Rhizoma atractylodes*	Atractylodine	14
*Atractylodes macrocephala*	Biatractylolide	10.5
*Poria cocos*	Pachymaran	10.5
*Coptis chinensis Franch*	Berberine	4
Maltose	Maltose	21
Total		100
**JSS prescription**
*Nepeta cataria L*.	*Nepeta cataria* oil	16.5
*Radix saposhnikoviae*	Chromone glycoside	16.5
*Notopterygium incisum*	Notopterol	16.5
Radix angelicae pubescentis	Heraclenin	16.5
Radix bupleuri	Saikosaponin	10
*Radix peucedani*	Peucedanin	10
*Poria cocos*	Pachymaran	10
*Glycyrrhiza uralensis*	Glycyrrhizin	4
Total		100

a Main active constituents of TCM come from Chinese pharmacopeia (2005).

### Sample collection

Blood samples were collected from the jugular vein using EDTA tubes for hematological analysis and flow cytometry. Piglets were euthanized by intravenous injection of sodium pentobarbital 40 mg/kg·BW. The abdominal cavity was then opened, and the spleen tissue was immediately harvested. The spleen index was calculated following the formula: spleen index = weight of spleen (g)/body weight (kg). Small pieces of spleen tissue were fixed in 4% formaldehyde solution until hematoxylin and eosin (H&E) staining. All remnant spleen tissues were kept frozen at −80°C until needed.

### H&E staining

The spleen tissues were taken from 4% formaldehyde solution and embedded in paraffin. The paraffin sections were then deparaffinized in xylene and rehydrated in a high-ethanol-to-water gradient (from 70 to 100%). Following that, sections were stained with hematoxylin and differentiated with 1% HCl in 70% alcohol. The sections were stained with eosin after being rinsed with tap water, then dehydrated and differentiated as described above. Finally, the sections were washed with xylene and mounted with neutral resin. To observe the histopathological changes of the spleen, the sections were examined under a light microscope. The spleen damage score was performed using a blinded fashion method according to previous studies (20, 21). The scoring rules are as follows: normal structure (0 point), granule denaturation (1 point); vacuolar degeneration (2 points), and apoptotic or necrosis (3 points).

### Hematological analysis

Hematology was performed in Ethylene Diamine Tetraacetic Acid (EDTA) full blood by automatic whole blood count (BC-5000 Vet, Mindray, China). The levels of white blood cells (WBC), Lym, monocytes (Mon), neutrophil (Neu), eosnophils (Eos) and basophil (Bas), red blood cell (RBC), hemoglobin (HGB), hematokrit (HCT), mean corpuscular volume (MCV), mean corpuscular hemoglobin (MCH), MCH concentration (MCHC), and platelets (PLT) were analyzed.

### Flow cytometric analysis

Peripheral blood single cell suspensions of lymphocytes and flow cytometric were performed as described in previous studies (22, 23). For T-cell phenotyping, blood samples were double-stained with monoclonal antibodies for CD4 (PE-Cy™7 Mouse Anti-Pig CD4, BD, USA) and CD8 (APC Mouse Anti-Pig CD8, BD, USA) or the corresponding isotype controls. The ratio CD4+/CD8+ was calculated based on the relative percentage of CD4+ and CD8+.

### ELISA kit assay

The levels of inflammatory markers in the spleen were measured using the Interleukin (IL)-1β (Beijing Chenglin Biological Technology Co., LTD., China, AD0125Po), Porcine IL-6 (Beijing Chenglin Biological Technology Co., LTD., China, AD0120Po), Porcine IL-8 (Beijing Chenglin Biological Technology Co., LTD., China, AD0056Po), and tumor necrosis factor alpha (TNF-α, Beijing Chenglin Biological Technology Co., LTD., China, AD0070Po) ELISA assay kits according to the manufacturer's protocol, respectively.

### Quantitative real-time polymerase chain reaction

Quantitative real-time polymerase chain reaction (qRT-PCR) was used to determine the mRNA expression of inflammation and antioxidant-related genes. Briefly, the total RNA was isolated using the TransZol Reagent (TransGen Biotech, Beijing, China). The complementary DNA (cDNA) was synthesized using a *TransScript*^®^ One-Step gDNA Removal and cDNA Synthesis SuperMix reagent kit (TransGen Biotech, Beijing, China) according to the kit's instructions. Then, it was stored at −20°C for SYBR Green qRT-PCR. All primer sequences were designed by OLIGO7 and presented in Supplementary Table 2. Relative gene expression levels were assessed using the 2^−Δ*ΔCT*^ method and normalized to the housekeeping genes GAPDH and β-actin.

### Western blot

Total protein of spleen was extracted with RIPA Lysis buffer. The details of Western Blot were consistent with our previous (9). The blots were semi-quantified using Image J software. The primary antibodies for NF-κB p65 (Cell Signaling, 6956, 1:1,000), phospho-NF-κB p65 (p-NF-κB p65, Cell Signaling, 3033, 1:1,000), inhibitor of NF-κBα (IκB-α, Cell Signaling, 4814, 1:1,000), phospho-IκB-α (p-IκB-α, Cell Signaling, 9246, 1:1,000), Nrf2 (Proteintech Group, 16396-1-AP, 1:1,000), Heme oxygenase-1 (HO-1, Proteintech Group, 27282-1-AP, 1:1,000) and superoxide dismutase 2 (SOD2, Abclonal, A19576, 1:1,500), and the HRP-conjugated secondary antibody (Bioss, bs-40295G-HRP, 1:3,000) were used in this study.

### Statistical analysis

All data in this study were presented as the mean ± standard deviation (SD). One-way analysis of variance was used for the statistical analysis, and then Tukey's multiple comparison test was performed with SPSS software (SPSS Inc., Chicago, IL, USA). Principal component analysis (PCA) and network analysis and correlation analysis were performed using WEKEMO Bioincloud online software (https://www.bioincloud.tech/). The statistical difference was considered significant at *P* < 0.05 and highly significant at *P* < 0.01, respectively.

## Results

### XJZ and JSS prescriptions ameliorated spleen morphological damage in piglets

**Figure 1:**
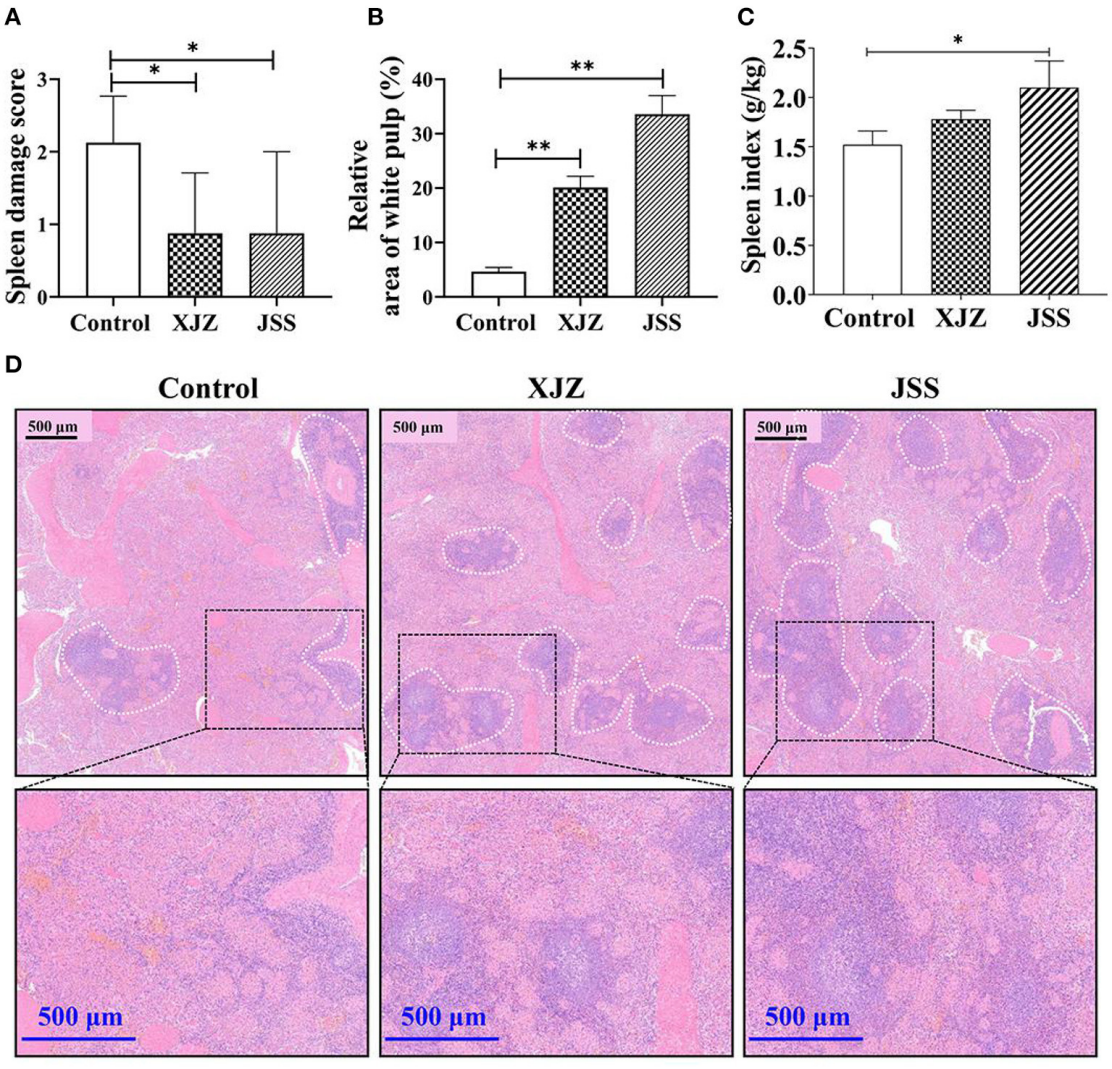
Effect of XJZ and JSS prescriptions on spleen health in weaned piglets. *P < 0.05 and **P < 0.01 vs. the control group. Data are expressed as means ± SD. **(A)** Spleen damage score. Blind scoring based on HE-stained sections. **(B)** Relative area of white pulp in the spleen. **(C)** Spleen index. Ratio of spleen weight to animal weight. **(D)** H&E staining imaging of spleen tissues in weaned-piglets. The white dotted line marks the white pulp area of the spleen.

To confirm the potential ameliorative effects of XJZ and JSS on spleen microstructure, H&E staining was performed (Figure 1). As shown in Figure 1D, in both XJZ and JSS groups, the splenocytes were tightly and adequately arranged with distinct nuclei and boundary between the red and white medulla, whereas its boundary in the control group were blurred. Notably (Figure 1A), the spleen damage score was significantly reduced in both TCM groups (*P* < 0.05). Surprisingly, the relative area of white pulp was markedly elevated after XJZ and JSS treatment (*P* < 0.01). Consistent with this result, there a marked increase of spleen index was observed after JSS treatment (*P* < 0.05). These results indicated that TCM prescriptions, XJZ and JSS, could ameliorate the histological structure of spleen and alleviated its morphological damage induced by weaning in piglets.

### XJZ and JSS prescriptions increased monocytes numbers in peripheral blood

To evaluate the effect of TCM on blood parameters, a blood routine test was performed on the peripheral blood of weaned piglets. As presented in Table 2, all blood parameters in the Control, XJZ and JSS groups were within the physiological range. Compared to the Control group, supplementation with XJZ and JSS was no significant (*P* > 0.05) difference on WBC, Lym, Neu, Eos, Bas, RBC, HGB, HCT, MCV, MCH, MCHC, and PLT. The levels of Mon in the control group were the lowest, which was significantly lower than those in the XJZ and JSS group (*P* < 0.05). These results suggested that the supplementation with XJZ and JSS is safe for weaned piglets at the doses used in this study and they have the potential to improve immunity.

**Table 2:** Effects of XJZ and JSS prescriptions supplement on hematological variables of piglets.

**Items**	**Groups**		
	**Control**	**XJZ**	**JSS**
WBC, 10^9^/L	16.01 ± 2.38	15.89 ± 2.53	17.57 ± 2.10
Lym, 10^9^/L	9.92 ± 1.65	9.57 ± 1.26	10.42 ± 1.38
Lym (%)	58.64 ± 10.11	61.99 ± 7.96	61.18 ± 5.33
Mon, 10^9^/L	1.06 ± 0.48	1.25 ± 0.50	1.18 ± 0.18
Mon (%)	5.6 ± 1.13	6.99 ± 1.36*	6.73 ± 1.07*
Neu, 10^9^/L	5.91 ± 1.83	5.98 ± 1.43	6.02 ± 1.57
Neu (%)	33.34 ± 5.95	36.54 ± 9.03	32.43 ± 7.15
Eos, 10^9^/L	0.48 ± 0.16	0.48 ± 0.21	0.58 ± 0.17
Eos (%)	2.71 ± 0.78	2.67 ± 1.42	3.38 ± 0.89
Bas, 10^9^/L	0.14 ± 0.03	0.18 ± 0.05	0.15 ± 0.03
Bas (%)	0.80 ± 0.10	0.95 ± 0.09	0.87 ± 0.22
RBC, 10^12^/L	5.79 ± 0.35	5.87 ± 0.43	5.94 ± 0.43
HGB, g/L	108.00 ± 8.44	101.30 ± 6.58	106.90 ± 9.35
HCT (%)	31.86 ± 2.50	30.44 ± 1.71	31.73 ± 2.69
MCV (fl)	55.03 ± 2.32	54.01 ± 2.95	53.39 ± 2.30
MCH (pg)	18.67 ± 0.84	17.40 ± 0.93	17.83 ± 1.02
MCHC (g/L)	339.33 ± 6.81	332.30 ± 6.43	337.10 ± 5.04
PLT, 10^12^/L	351.50 ± 39.44	393.87 ± 75.69	326.00 ± 87.46

WBC, white blood cells; Lym, lymphocyte; Mon, monocytes; Neu, neutrophil; Eos, eosnophils; Bas, baosphil; RBC, red blood cell; HGB, hemoglobin; HCT, hematokrit; MCV, mean corpuscular volume; MCH, mean corpuscular hemoglobin; PLT, platelets. Results are expressed as mean ± SD, one-way ANOVA, n = 6. ^*^P <0.05 vs. the control group.

### XJZ and JSS prescriptions improved the Lym subpopulations CD4+/CD8+ ratio

**Figure 2:**
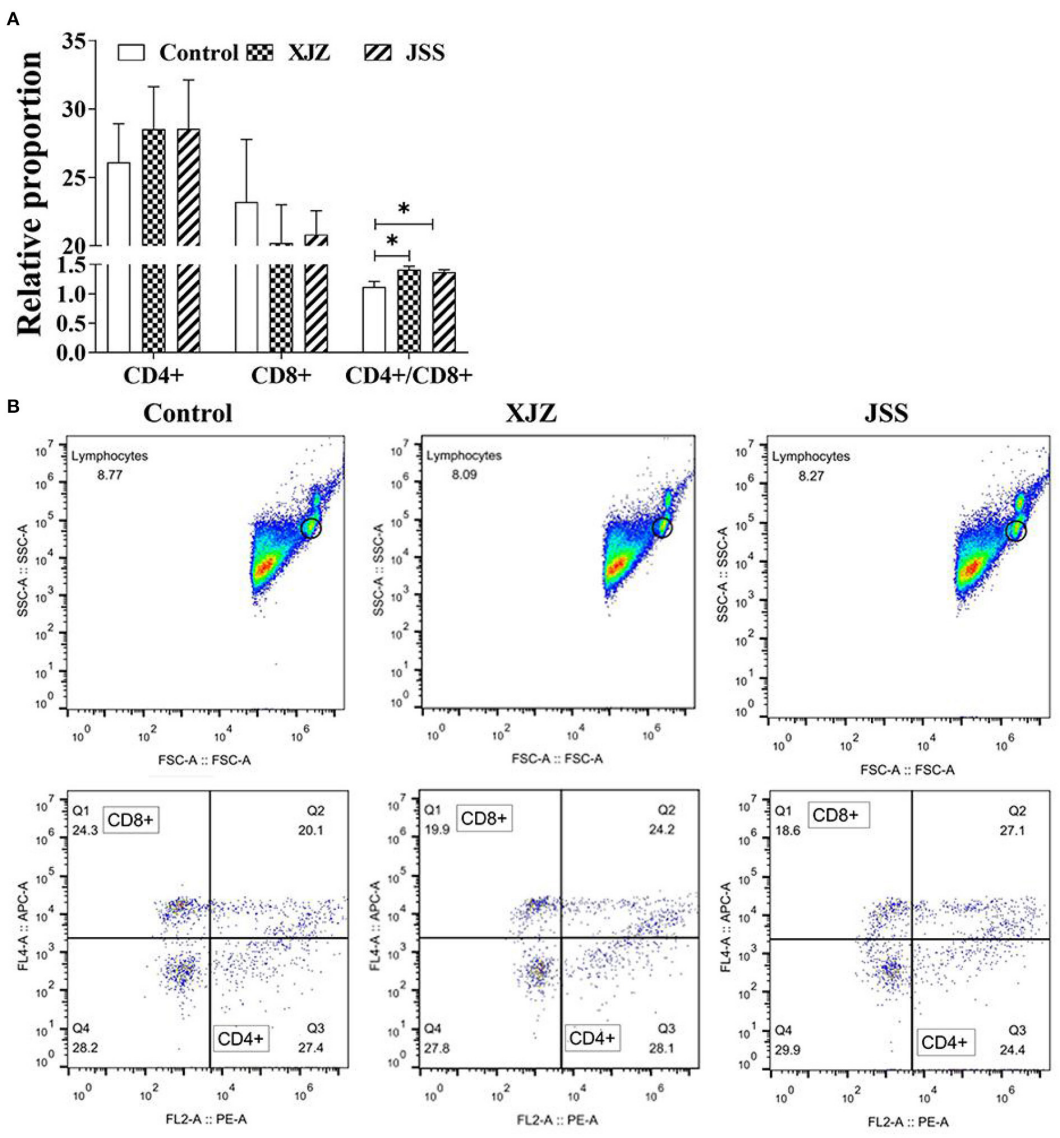
Effects of XJZ and JSS prescriptions on proportion of CD4+ and CD8+ subpopulations in peripheral blood of piglets. *P < 0.05 vs. the control group. Data are expressed as means ± SD. **(A)** Statistical analysis of Lymphocyte Subpopulations CD4+ and CD8+. **(B)** Flow cytometry analysis of CD4+ and CD8+.

To explore the effect of TCM on immune performance, we analyzed the levels of Lym subpopulations CD4+ and CD8+. As shown in Figures 2A,B, there was no apparently difference in the relative proportions of CD4+ and CD8+ subpopulations (*P* > 0.05). Surprisingly, both XJZ and JSS significantly increased the ratio of CD4+/CD8+ (*P* < 0.05). These results showed that both TCM treatments could improve immune performance by increasing the ratio of CD4+/CD8+.

### XJZ and JSS prescriptions reduced the levels of inflammatory marker in the spleen

Activation of inflammatory response is a hallmark of stress-induced spleen damage in piglets. As shown in Table 3, both TCM prescriptions obviously reduced the inflammatory markers level of IL1β (*P* < 0.01), IL6 (*P* < 0.01), IL8 (*P* < 0.01 or *P* < 0.05), and TNF-α (*P* < 0.01). Hence, there is no doubt that XJZ and JSS prescriptions have anti-inflammatory potential.

**Table 3:** Effect of XJZ and JSS prescriptions on spleen inflammatory markers of weaned piglets.

**Item**	**Con**	**XJZ**	**JSS**
IL-1β, ng/L	88.32 ± 4.98	74.33 ± 5.54**	76.05 ± 4.83**
IL-6, ng/L	45.74 ± 3.69	36.88 ± 2.85**	36.89 ± 2.63**
IL-8, ng/L	71.10 ± 4.29	58.55 ± 3.45**	65.34 ± 3.51*
TNF-α, ng/L	79.25 ± 5.82	65.44 ± 5.16**	66.42 ± 4.88**

IL-1β, interleukin-1β; IL-6, interleukin-6; IL-8, interleukin-8; TNF-α, tumor necrosis factor-alpha. Results are expressed as mean ± SD, one-way ANOVA, n = 6. ^*^P <0.05 and ^**^P <0.01 vs. the control group.

### XJZ and JSS prescriptions inhibited NF-κB signaling pathway

**Figure 3:**
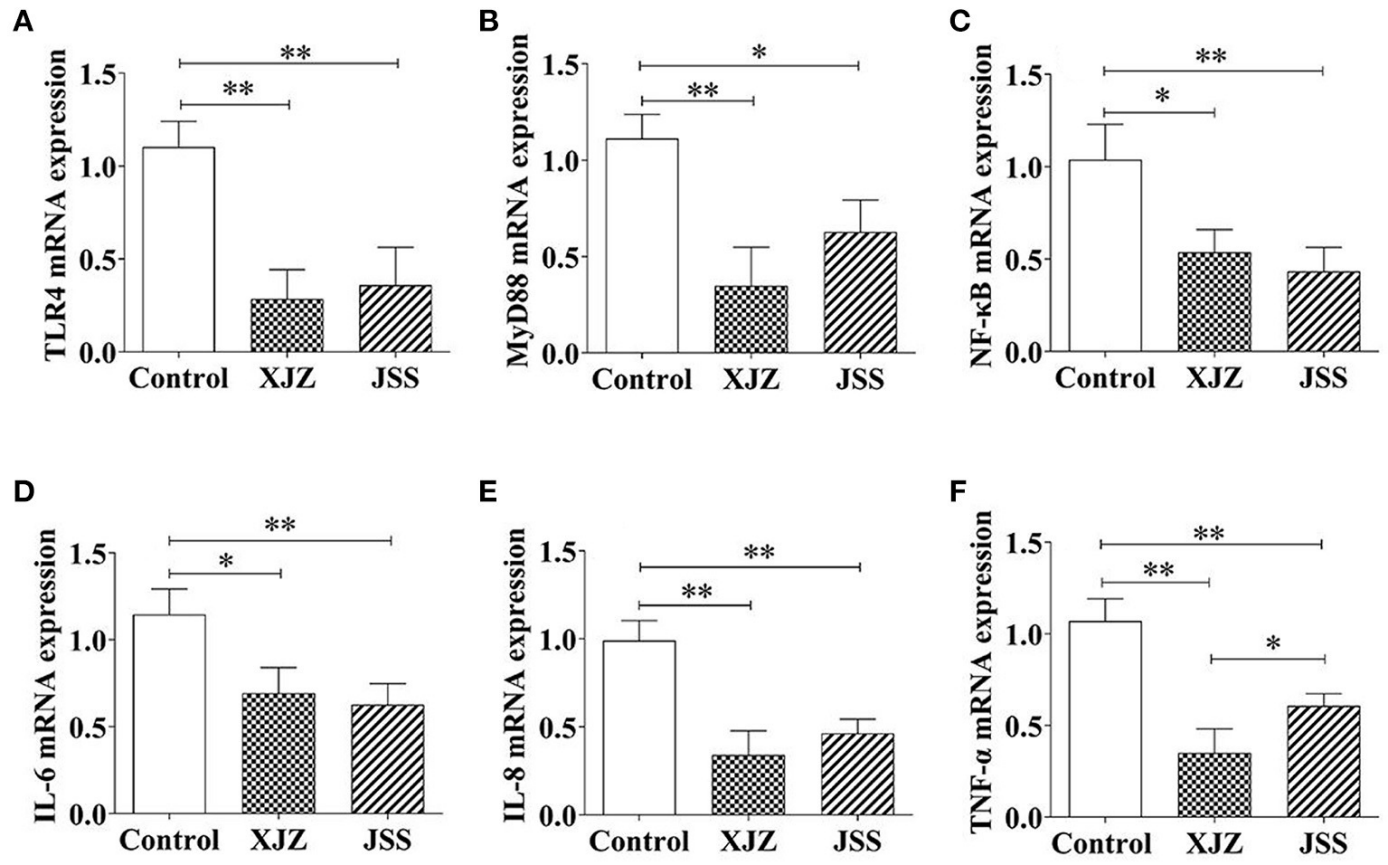
Effects of XJZ and JSS prescriptions on mRNA levels of NF-κB signaling pathway in the spleen of weaned-piglets. *P < 0.05 and **P < 0.01 vs. the control group. Data are expressed as means ± SD. **(A)** TLR4, **(B)** MyD88, **(C)** NF-κB, **(D)** IL-6, **(E)** IL-8, and **(F)** TNF-α.

**Figure 4:**
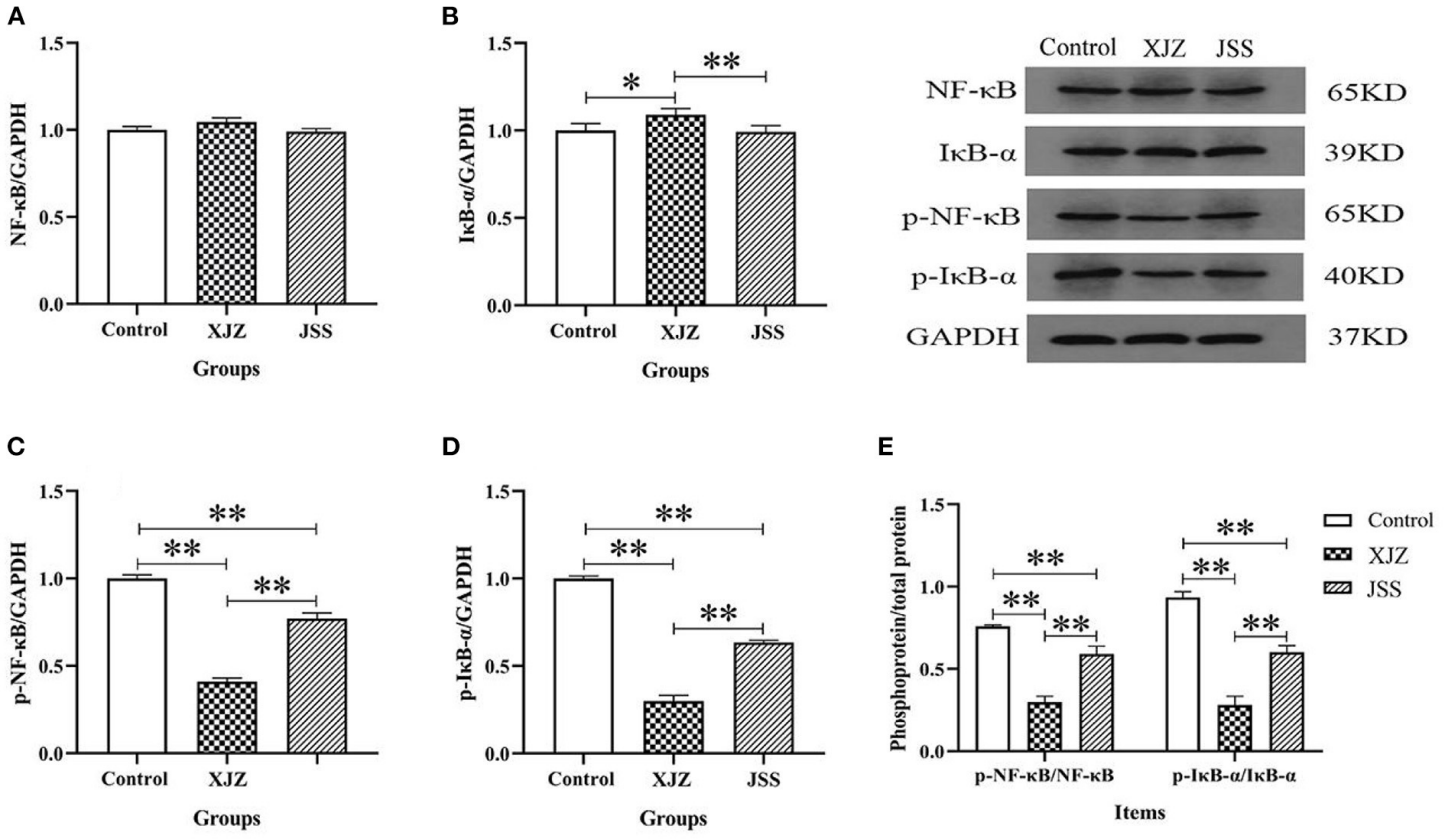
Effect of both XJZ and JSS prescriptions on protein level of NF-κB signaling pathway in the spleen of weaned-piglets. *P < 0.05 and **P < 0.01 vs. the control group. Data are expressed as means ± SD. **(A)** NF-κB/GAPDH, **(B)** IκB-α/GAPDH, **(C)** p-NF-κB/GAPDH, **(D)** p-IκB-α/GAPDH, and **(E)** Phosphoprotein/total protein.

NF-κB is a key regulator of inflammation and it is involved in both innate and adaptive immune responses. Our results showed that both TCM treatments conspicuously suppressed the mRNA expression of pattern recognition receptor (Toll-like Receptor 4, TLR4, *P* < 0.01, Figure 3A) and adapter protein (myeloid differentiation primary response 88, Myd88, *P* < 0.01 or *P* < 0.05, Figure 3B). Likewise, the NF-κB transcription level also significantly decreased as expected after XJZ (*P* < 0.05) and JSS (*P* < 0.01) treatment (Figure 3C). Moreover, the mRNA levels of NF-κB downstream pro-inflammatory factors, IL-6 (*P* < 0.05 or *P* < 0.01) and IL-8 (*P* < 0.01) were also prominently decreased (Figures 3D–F). At the protein level, there was no significant change in the total protein level of NF-κB in the XJZ and JSS groups (Figure 4A). As an inhibitor of NF-κB, the levels of IκB-α were markedly increased (*P* < 0.05) after XJZ treatment (Figure 4B). Importantly, the protein levels of p-NF-κB (Figure 4C) and p-IκB-α (Figure 4D) were notably decreased in the spleen after XJZ and JSS supplementation (*P* < 0.01). Furthermore, both TCM prescriptions, XJZ and JSS, remarkably reduced the ratio of p-NF-κB/NF-κB and p-IκB-α/IκB-α (*P* < 0.01, Figure 4E). These results revealed that XJZ and JSS could relieve inflammatory response of spleen in weaned piglets *via* TLR4-Myd88-NF-κB dependent pathway.

### XJZ and JSS prescriptions activated antioxidant system of spleen in weaned-piglets

**Figure 5:**
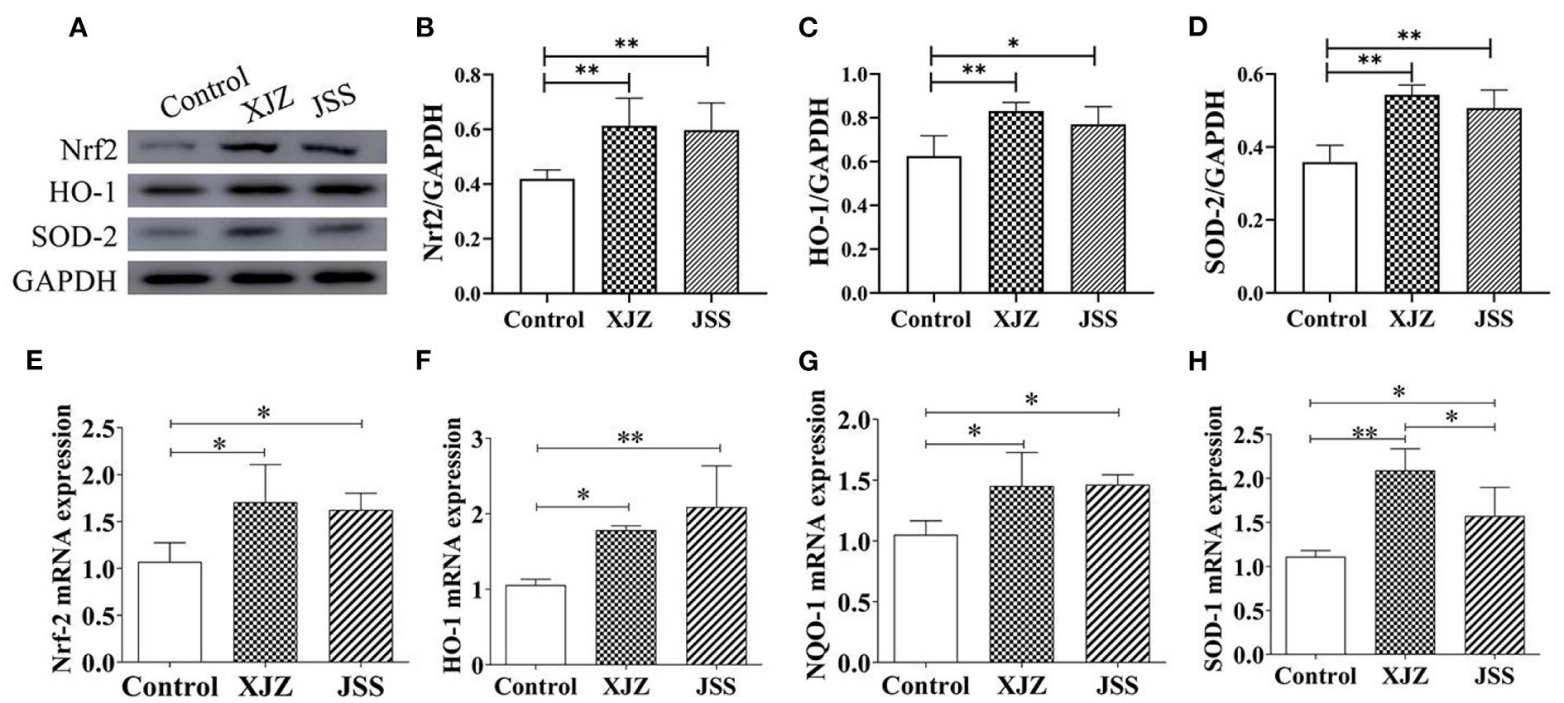
Effects of XJZ and JSS prescriptions on antioxidant defense system of spleen in weaned piglets. *P < 0.05 and **P < 0.01 vs. the control group. Data are expressed as means ± SD. **(A)** Bands of Nrf2, HO-1, SOD-2. **(B)** Nrf2/GAPDH. **(C)** HO-1/GAPDH. **(D)** SOD-2/GAPDH. **(E)** Nrf2 mRNA expression. **(F)** HO-1 mRNA expression. **(G)** NQO-1 mRNA expression. **(H)** SOD-1 mRNA expression.

As a therapeutic target for many diseases and damage, Nrf2 is a switch on the endogenous antioxidant defense system. As shown in Figures 5A–D, the protein levels of Nrf2 (*P* < 0.01) and antioxidases (HO-1, *P* < 0.05 or *P* < 0.01 and SOD 2, *P* < 0.01) were significantly upregulated in both TCM groups. Likewise, both XJZ and JSS also markedly increased the gene expression of Nrf2 (*P* < 0.05, Figure 5E) HO-1 (*P* < 0.05 or *P* < 0.01, Figure 5F) NQO1 (*P* < 0.05, Figure 5G) and SOD-1 (*P* < 0.05 or *P* < 0.01, Figure 5H). These results indicated that XJZ and JSS prescriptions could promote the spleen antioxidant capacity in weaned piglets by stimulating Nrf2 antioxidant defense response.

### XJZ and JSS prescriptions alleviated spleen damage *via* crosstalk of NF-κB and Nrf2

**Figure 6:**
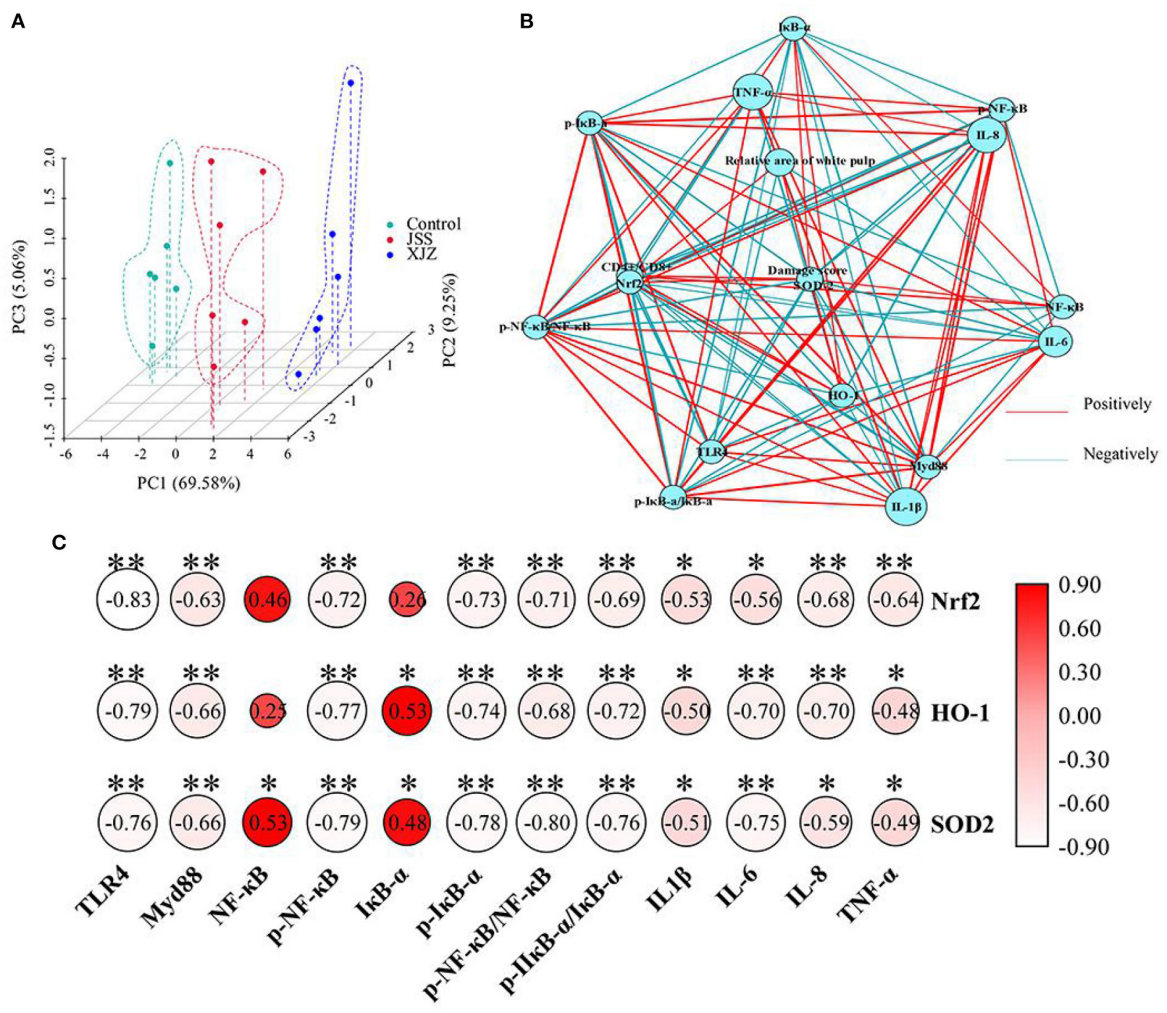
Effects of XJZ and JSS prescriptions on the interaction of inflammation-related and antioxidant-related molecules in the spleen of weaned piglets. **(A)** Principal component analysis (PCA) of inflammation-related and antioxidant-related molecules expression. **(B)** Network analysis of spleen damage score, inflammation/antioxidant-related molecules. The threshold for the absolute value of the correlation coefficient is 0.4. **(C)** Correlation analysis between NF-κB-mediated inflammatory pathway and Nrf2-mediated antioxidant defense system. *P < 0.05 and **P < 0.01 vs. the control group.

To determine the role of NF-κB and Nrf2 signaling pathways in TCM prescription ameliorating spleen damage, the PCA, network and correlation analysis were applied. As shown in Figure 6A, there was no overlap among these groups, indicating that the expression profiles of inflammation-related genes and antioxidant-related factors were significantly changed after XJZ and JSS treatment. Network analysis (Figure 6B) showed that the damage score and SOD2 were located at the center, suggesting that they are associated with the expression of multiple factors. It is worth emphasizing that there was a markedly (*P* < 0.05 or *P* < 0.01) positive correlation between the damage score and the levels of p-NF-κB/NF-κB, p-IκB-α/IκB-α, IL-1β, and IL-6. Moreover, an obvious negative correlation was observed between the damage score and the expression of HO-1, and SOD2 (*P* < 0.05 or *P* < 0.01). Of note, correlation analysis revealed that there was a crosstalk between inflammatory signaling pathway and antioxidant defense system, (Figure 6C) as reflected by a significant (*P* < 0.05 or *P* < 0.01) correlation between antioxidant molecules (Nrf2, HO-1, SOD2) and NF-κB signaling pathway (TLR4, Myd88, p-NF-κB, p-IκB-α, p-NF-κB/NF-κB, p-IκB-α/IκB-α, IL-1β, IL-6, IL-8, and TNF-α). These results suggested that XJZ and JSS intervention may alleviate spleen injury in weaned piglets *via* the crosstalk of NF-κB-mediated inflammatory signaling pathway and Nrf2-mediated antioxidant defense system.

## Discussion

For thousands of years, TCM prescription has contributed to human health in China and Asian countries. The compatibility of prescription drugs is strictly in accordance with the theory of TCM. It is well-known that most of TCM are derived from natural plants or minerals, and the pharmaceutical activities and therapeutic effects of these natural ingredients have been proven and witnessed in long-term clinical applications (24). XJZ and JSS prescriptions have been used in China for hundreds of years, mainly for the treatment of organ function injury and body fatigue. The main ingredients of the XJZ prescription include *Cassia Twig* (13%), *Cynanchum otophyllum* (13%), *Rhizoma Atractylodes* (14%), *Atractylodes macrocephala* (10.5%), *Poria cocos* (10.5%), Maltose (21%). Among these materials, the main effect of maltose is to improve the palatability of the prescription, and *Cassia Twig* (25), *Cynanchum otophyllum* (26), *Rhizoma Atractylodes* (27) have been shown to have anti-inflammatory and antiviral properties, and extracts of *Atractylodes macrocephala* (28), *Poria cocos* (29) exert antioxidant properties. In JSS prescription, the main components, *Nepeta cataria L., Radix Saposhnikoviae, Notopterygium incisum, and Radix Angelicae pubescentis* have an extensive range of biological activities, including antimicrobial, antioxidant and anti-inflammatory, as well as anti-ulcer and insecticidal properties (13, 30, 31). Due to the complex pathogenic factors and various diseases caused by weaning stress, the clinical effect of monotherapy may not meet expectations (1). In addition, Western medicine is increasingly inclined to use multiple drugs to synergistically treat some multi-organ damage diseases and immunosuppressive diseases, such as AIDS (32). Weaning stress induces oxidative damage (33), diarrhea (9), multi-organ inflammation (34), and reduced immune function in piglets. Therefore, the use of TCM treatment has unparalleled advantages.

The spleen is an immune organ innervated by sympathetic nerves, which together with the adrenomedullary system control splenic immune functions (35). In addition, as an important lymph organ, the spleen is the center of cellular immunity and humoral immunity, which can exert anti-inflammatory and antiviral effects by regulating lymphoid tissues and immune-related cytokines (36). Meanwhile, the structure and function of the spleen is comprehensive and is involved in digestion, absorption, energy conversion, and immune regulation. Based on the above concept, for weaned-piglets, with immature digestive and immune systems, maintaining or improving the spleen structure and function is critical. Study indicated that the immune state of organisms can be reflected by the spleen index (37, 38). In our study, JSS notably elevated the spleen index of piglets, meanwhile, both XJZ and JSS groups ameliorated the microstructure of spleen, and alleviated the morphological damage induced by weaning stress. Notably, the relative area of white pulp was increased after supplementation of XJZ and JSS. The white pulp and the red pulp are two distinct parts of the spleen's anatomy. Lymphoid tissue, mostly lymphocytes (T cells and B cells) and macrophages, make up the white pulp. Thus, the promotion in the white pulp and spleen index suggested an increase in the immune function of the spleen. In line with Lee et al. (22) reported that exposure of spleen cells to a turmeric extract (zingiberaceae, similar to *zingiber officinale* roscoe) *in vitro* elevated the proliferation of lymphocyte. Speculatively, the promotion of lymphocyte proliferation by certain active ingredients can be linked to the increase in the region of white pulp in the spleen. Importantly, the results of peripheral blood Mon proportion and CD4+/CD8+ ratio of T-lymphocytes also supported the enhancement of immune function. As the largest white blood cell, Mon plays a vital role in immune response, phagocytosis, and clearing injured senescent cells or its debris (39). Furthermore, CD4+ and CD8+ T cells are the central factors of immune regulation and immune response. The CD4+/CD8+ ratio was significantly increased after treatment with XJZ and JSS prescriptions, suggesting that the blood immunity of piglets in both XJZ and JSS groups were improved. In other words, the susceptibility to the disease was reduced. The ameliorative effect of TCM prescription on the blood immunity of piglets may be related to the enhancement of immune response activity by some of their active ingredients. For example, in chickens, the expression of a family of innate immune response genes were up-regulated when supplemented with cinnamaldehyde in the diet (40). Moreover, our previous study found that both XJZ and JSS prescriptions could improve the growth rate and reduce the rate of diarrhea in piglets (9). Therefore, these results suggested that XJZ and JSS could promote the growth performance, control diarrhea induced by weaning stress, and improve the blood immune-related factors level, which may be related to maintaining the spleen function of weaned piglets.

Inflammatory and oxidative damage induced by weaning or other stress are the key factors contributing to the decline of spleen physiological function in piglets (15). NF-κB is a nuclear transcription factor that is activated by pattern recognition receptors and mediates the splenic inflammatory response. TLR4 is the first pattern recognition receptors discovered, which can activate Myd88 and NF-κB, thereby promoting the release pro-inflammatory cytokines and gene transcription (29). An earlier study showed that the TCM extract reduced proinflammatory cytokines, reduced oxidative stress, and boosted antioxidant protection system in a mouse model of sepsis, preventing spleen and liver injury (41). Similarly, Rao et al. demonstrated that, in LPS-stimulated RAW264.7 cells, JSS prescription exerted precise anti-inflammatory effects that are regulated by suppression of the NF-κB signaling pathway (13). In our study, both TCM groups apparently reduced pro-infammatory factors level of IL-1β, IL-6, IL-8, and TNF-α in the spleen, consequently verifying the anti-inflammatory effect of both XJZ and JSS. Moreover, excessive release of inflammatory factors causes cytokine imbalance and immune dysfunction, further injuring the spleen function. The activation of the NF-κB inflammatory signaling pathway is linked to the production of these inflammatory cytokines. Consistent with Xu et al. (42) findings, both XJZ and JSS treatments reduced TLR4, MyD88, and NF-κB mRNA levels, together with the phosphorylated protein of p-NF-κB and p-IκB-α. These results suggested that XJZ and JSS prescriptions targeted the NF-κB inflammatory signaling pathway and ameliorated weaning-induced splenic inflammatory injury. In addition, the inhibition of inflammatory response may be related to the activation of spleen antioxidant defense system by TCM prescription.

Oxidative damage occurs when biomolecules such as lipids, DNA, and proteins are oxidized, resulting in reduced functionality of basic physiological processes. Additionally, oxidative stress is often associated with inflammation. As ulcerative colitis (UC) strikes, for example, a vast of Lym and macrophages are activated; these cells then migrate to the damaged mucous membrane, where they produce an excessive amount of oxygen free radicals, damaging or even killing the inflammation region (43). Oxidative stress activates molecular pathways that trigger inflammation and can directly contribute to spleen damage. This type of tissue injury is considered one of the most significant mechanisms leading to organ dysfunction. As previous study showed, weaning and other stress induced oxidative damage to multiple organs in piglets, such as the hepatic and intestine (33). The Nrf2 signaling pathway, as one of the most essential defense systems, is a vital path to stimulate the expression of antioxidants and phase II enzymes. Meanwhile, many natural compounds exert their activity through it (44). Nrf2 is a transcription factor that usually found in the cytoplasm as a dormant protein tethered to Keap1. By binding to ARE, Nrf2 activates the expression of phase II enzymes, such as HO-1 and NQO1, once it translocates from the cytoplasm to the nucleus in response to oidative signals. Liu et al. (45) found that soyasaponin, a natural active ingredient derived from plant, provided its defense effect of anti-oxidative stress *via* Nrf2 signaling pathway. Likewise, Li et al. (46) reported that the active ingredients in TCM, such as flavonoids, triterpenoid saponins, quinones, terpenoids, and phthalide class, can improve the body's antioxidant capacity by activating the Nrf2 signaling pathway. Moreover, these active ingredients, such as triterpenoid saponins and terpenoids, were included in both XJZ and JSS prescriptions. In our study, we observed that both TCM prescription treatments prominent increased antioxidant-related gene levels of Nrf-2, HO-1, NQO-1, and SOD-1, which suggested that XJZ and JSS prescriptions could ameliorate the antioxidant capacity and alleviate the oxidative damage of spleen by activating the Nrf2 signaling pathway. Meanwhile, the higher Nrf2, HO-1 and SOD2 protein levels of TCM treatments also supported it. Therefore, based on the above results, both XJZ and JSS prescriptions have potential protective effects on oxidative injury in the spleen of piglets by activating Nrf2-mediated endogenous antioxidant system. Concomitantly, according to the results of PCA and network analysis, we found the spleen damage scores was at the center of the network, suggested that it correlates with the expression of multiple molecules. Surprisingly, there was a significant correlation between NF-κB-mediated inflammatory signaling axis and Nrf2-mediated antioxidant defense system, which suggested that both XJZ and JSS intervention may alleviate spleen damage by crosstalk of NF-κB and Nrf2 signaling pathways.

**Figure 7:**
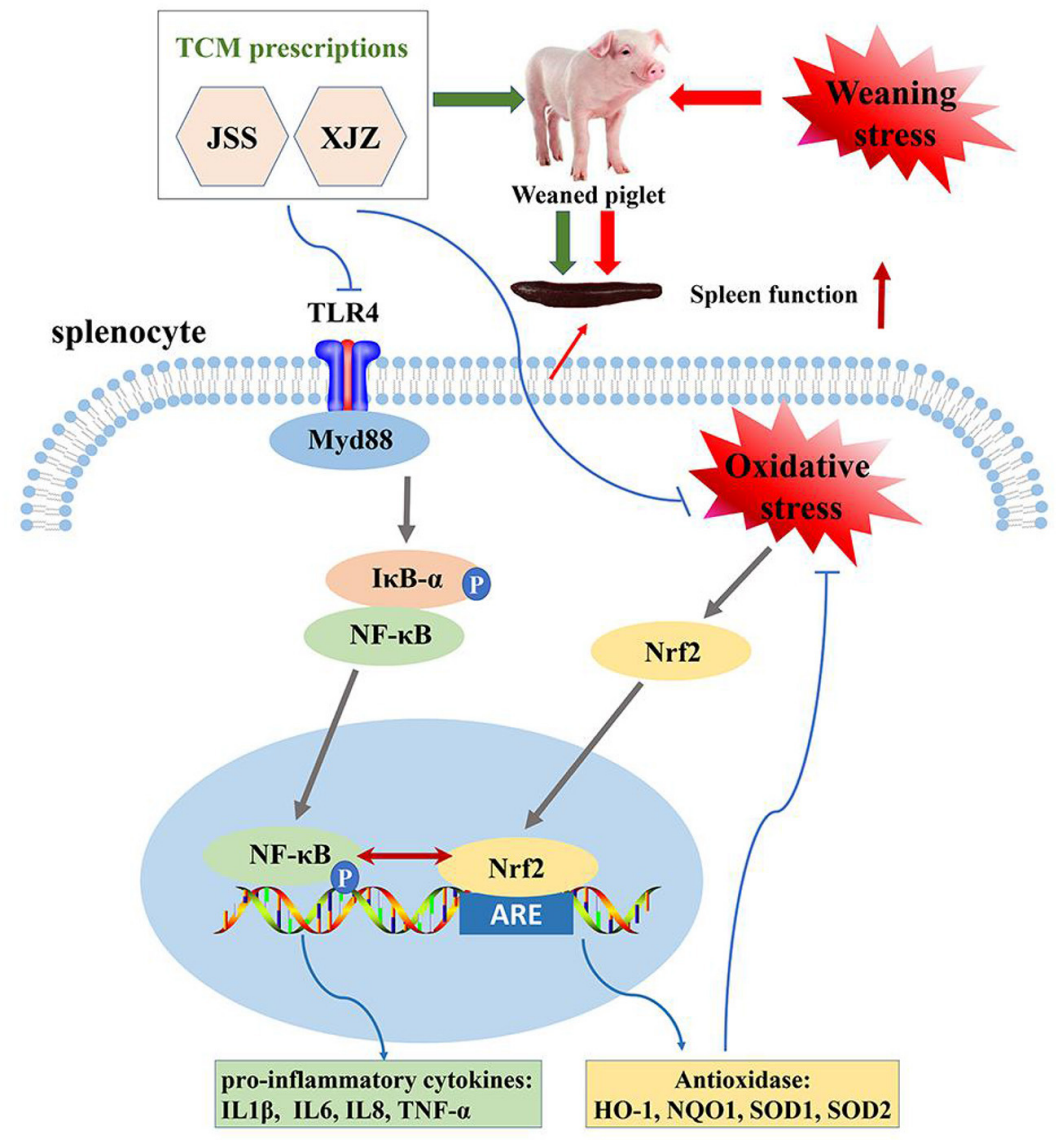
Schematic diagram illustrating the proposed mechanism of XJZ and JSS ameliorates spleen inflammatory response and antioxidant capacity by synergistically regulating NF-κB and Nrf2 signaling pathways in weaned-piglets.

In conclusion, our study found that both XJZ and JSS prescriptions ameliorated spleen antioxidant capacity and inflammatory response by synergistically regulating NF-κB and Nrf2 signaling pathways in weaned-piglets (Figure 7). Our study provides a new and effective strategy to antagonism immune weakening.

## Data availability statement

The datasets presented in this study can be found in online repositories. The names of the repository/repositories and accession number(s) can be found in the article/Supplementary material.

## Ethics statement

The animal study was reviewed and approved by the Institutional Animal Care and Use Committee of JiangXi Agricultural University.

## Author contributions

JC, NH, and HC designed the study. YM, AHu, WJ, AHua, YW, and PM performed the experiments. JC and NH analyzed the data. JC and HC drafted the article. NH, MH, and FY revised the article and rewrote the discussion. XY did the final review. YC checked the language of the article. HC administrated and supervised the study. All authors contributed to the article and approved the submitted version.

## Funding

This study was supported by major research and development projects of Jiangxi province (20194ABC28008).

## Conflict of interest

Author XY was employed by Jiangxi Zhongchengren Pharmaceutical Co., Ltd. Author YC was employed by Jiangxi Jiabo Biological Engineering Co., Ltd. The remaining authors declare that the research was conducted in the absence of any commercial or financial relationships that could be construed as a potential conflict of interest.

## Publisher's note

All claims expressed in this article are solely those of the authors and do not necessarily represent those of their affiliated organizations, or those of the publisher, the editors and the reviewers. Any product that may be evaluated in this article, or claim that may be made by its manufacturer, is not guaranteed or endorsed by the publisher.
