# Supplementary material for: Traditional Chinese medicine prescriptions (XJZ, JSS) ameliorate spleen inflammatory response and antioxidant capacity by synergistically regulating NF-κB and Nrf2 signaling pathways in piglets
Source: Front Vet Sci. 2022 Sep 16;9:993018. doi: 10.3389/fvets.2022.993018 (PMC9525143; doi:10.3389/fvets.2022.993018)
Supplement: Supplementary file 1 [file Data_Sheet_1.docx]

Supplementary Material

Traditional Chinese Medicine Prescriptions (XJZ, JSS) Ameliorate Spleen Inflammatory Response and Antioxidant Capacity by Synergistically Regulating NF-κB and Nrf2 Signaling Pathways in Weaned-Piglets

Jian Chen^1†^, Nianqing Hu^1†^, Yaqing Mao^2^, Aiming Hu^3^, Wenjuan Jiang^4^, Aimin Huang^1^,Yun Wang^5^, Puyan Meng^6^, Mingwen Hu^1^, Xiaobin Yang^7^, Yuandong Cao^8^, Fan Yang^1^, Huabin Cao^1^

^1^Jiangxi Provincial Key Laboratory for Animal Health, Institute of Animal Population Health, College of Animal Science and Technology, Jiangxi Agricultural University, Nanchang, Jiangxi, P. R., China

^2^MOA Center for Veterinary Drug Evaluation, China Institute of Veterinary Drug Control, Beijing, China

^3^Jian Animal Husbandry and Veterinary Bureau, Jian, Jiangxi, P. R., China

^4^Animal Husbandry and Aquatic Products Technology Application Extension Office, Jiangxi Agricultural Technology Extension Center, Nanchang, Jiangxi, P. R., China

^5^Jiangxi biotech vocational college, Nanchang, Jiangxi, P. R., China

^6^Jiangxi Academy of Forestry, Nanchang, Jiangxi, P. R., China

^7^Jiangxi Zhongchengren Pharmaceutical Co., Ltd., Nanchang, Jiangxi, P. R., China

^8^Jiangxi Jiabo Biological Engineering Co., Ltd., Jiujiang, Jiangxi, P. R., China

^†^ These two are equal first authors.

***Correspondence:**

Huabin Cao,

Jiangxi Provincial Key Laboratory for Animal Health, Institute of Animal Population Health, College of Animal Science and Technology, Jiangxi Agricultural University, Nanchang, Jiangxi, P. R., China

Email: [chbin20020804@jxau.edu.cn](mailto:chbin20020804@jxau.edu.cn)

**Supplementary Table 1:** Composition of the basal diet (as-fed basis)

| Item | Ingredient, % | Nutrient composition^2^ | Content, (g/kg) |
| --- | --- | --- | --- |
| Corn | 43.3 | DE(Mcal/kg) | 3.35 |
| Soybean meal | 25.4 | Crude protein (%) | 21.51 |
| Soy protein isolate | 3.00 | Lysine (%) | 1.41 |
| Whey powder | 7.50 | Methionine + Cystine (%) | 0.81 |
| Soybean oil | 1.50 | Threonine (%) | 0.94 |
| Lactose | 10.0 | Calcium (%) | 0.86 |
| Stone powder | 0.75 | Total phosphorus (%) | 0.70 |
| Calcium hydrogen phosphate | 1.05 |  |  |
| 50% choline chloride | 0.10 |  |  |
| Lysine | 0.22 |  |  |
| L-methionine | 0.10 |  |  |
| L-threonine | 0.08 |  |  |
| Vitamin and mineral premix^1^ | 1.00 |  |  |
| Total | 100 |  |  |

^1^ Vitamin and mineral premix supplied per kilogram diet: vitamin A, 18,000 IU; vitamin D, 4,000 IU; vitamin E, 50 mg; vitamin K_3_, 4 mg; vitamin B_1_, 4 mg; vitamin B_2_, 10 mg; vitamin B_6_, 4 mg; vitamin B_12_, 30 μg; pantothenic acid, 30 mg; folic acid, 2 mg; biotin, 0.16 mg; Fe, 150 mg; Cu, 18 mg; Mn, 48 mg; Zn, 150 mg; I, 1.5 mg; and Se, 0.3 mg.

^2^ Values were calculated according to NRC (2012).

**Supplementary Table 2**: Primers used in this study

| Target | GenBank Number | Primers Sequence (5'-3') |
| --- | --- | --- |
| GAPDH | NM_001206359 | F: ACTCACTCTTCCACTTTTGATGCT |
|  |  | R: TGTTGCTGTAGCCAAATTCA |
| β-actin | XM_021086047.1 | F: AGAAGCTGTGATGGACGCAG |
|  |  | R: ACCCCTGGGAGTTGTACCTT |
| TLR4 | NM_001113039.1 | F: GCCATCGCTGCTAACATCATC |
|  |  | R: CTCATACTCAAAGATACACCATCGG |
| MyD88 | NM_001099923.1 | F: TGGTAGTGGTTGTCTCTGATGA |
|  |  | R: TGGAGAGAGGCTGAGTGCAA |
| NF-κB | NM_001048232.1 | F: CTCGCACAAGGAGACATGAA |
|  |  | R: ACTCAGCCGGAAGGCATTAT |
| IL-6 | NM_001252429.1 | F: TGGCTACTGCCTTCCCTACC |
|  |  | R: CAGAGATTTTGCCGAGGATG |
| IL-8 | NM_213867.1 | F: TTCGATGCCAGTGCATAAATA |
|  |  | R: CTGTACAACCTTCTGCACCCA |
| TNF-α | NM_214022.1 | F: CCAATGGGCAGAHTGGGTATG |
|  |  | R: TGAAGAGGACCTGGGAGTAG |
| Nrf2 | XM_005671982.1 | F: CCCATTCACAAAAGACAAACATTC |
|  |  | R: GCTTTTGCCCTTAGCTCATCTC |
| HO-1 | NM_001004027 | F: CGCTCCCGAATGAACAC |
|  |  | R: GCTCCTGCACCTCCTC |
| NQO1 | NM_001159613.1 | F: CCAGCAGCCCGGCCAATCTG |
|  |  | R: AGGTCCGACACGGCGACCTC |
| SOD-1 | NM_001190422.1 | F: GAGACCTGGGCAATGTGACT |
|  |  | R: CTGCCCAAGTCATCTGGTT |

F, forward; R, reverse; GAPDH, Glyceraldehyde-3-phosphate dehydrogenase; TLR4, Toll-like receptor 4; MyD88, Myeloid differentiation factor 88; NF-κB, Nuclear factor-κB; IL-6, Interleukin-6; IL-8, Interleukin-8; TNF-α, Tumor necrosis factor-α; Nrf2, Nuclear factor erythroid 2-related factor 2; HO-1, Heme oxygenase-1; NQO1, NAD(P)H quinone oxidoreductase-1; SOD-1, superoxide dismutase-1.
